# Relationship between Numerous Mast Cells and Early Follicular Development in Neonatal MRL/MpJ Mouse Ovaries

**DOI:** 10.1371/journal.pone.0077246

**Published:** 2013-10-04

**Authors:** Teppei Nakamura, Saori Otsuka, Osamu Ichii, Yuko Sakata, Ken-Ichi Nagasaki, Yoshiharu Hashimoto, Yasuhiro Kon

**Affiliations:** 1 Laboratory of Anatomy, Department of Biomedical Sciences, Graduate School of Veterinary Medicine, Hokkaido University, Sapporo, Japan; 2 Section of Biological Safety Research, Chitose Laboratory, Japan Food Research Laboratories, Chitose, Japan; 3 Office for Faculty Development and Teaching Enriched Veterinary Medicine, Graduate School of Veterinary Medicine, Hokkaido University, Sapporo, Japan; INSERM-Université Paris-Sud, France

## Abstract

In the neonatal mouse ovary, clusters of oocytes called nests break into smaller cysts and subsequently form individual follicles. During this period, we found numerous mast cells in the ovary of MRL/MpJ mice and investigated their appearance and morphology with follicular development. The ovarian mast cells, which were already present at postnatal day 0, tended to localize adjacent to the surface epithelium. Among 11 different mouse strains, MRL/MpJ mice possessed the greatest number of ovarian mast cells. Ovarian mast cells were also found in DBA/1, BALB/c, NZW, and DBA/2 mice but rarely in C57BL/6, NZB, AKR, C3H/He, CBA, and ICR mice. The ovarian mast cells expressed connective tissue mast cell markers, although mast cells around the surface epithelium also expressed a mucosal mast cell marker in MRL/MpJ mice. Some ovarian mast cells migrated into the oocyte nests and directly contacted the compressed and degenerated oocytes. In MRL/MpJ mice, the number of oocytes in the nest was significantly lower than in the other strains, and the number of oocytes showed a positive correlation with the number of ovarian mast cells. The gene expression of a mast cell marker also correlated with the expression of an oocyte nest marker, suggesting a link between the appearance of ovarian ? 4mast cells and early follicular development. Furthermore, the expression of follicle developmental markers was significantly higher in MRL/MpJ mice than in C57BL/6 mice. These results indicate that the appearance of ovarian mast cells is a unique phenotype of neonatal MRL/MpJ mice, and that ovarian mast cells participate in early follicular development, especially nest breakdown.

## Introduction

Mast cells (MCs) reside in most tissues and act as sentinel cells in both innate and adaptive immunity [1,2]. MCs also contribute to the pathogenesis of cancer, obesity, and diabetes as well as to immunological processes such as allergy and autoimmunity [3–6]. In rodents, MCs are classified into 2 distinct subpopulations, namely, connective tissue MCs (CTMCs) and mucosal MCs (MMCs). These cell types are distinguished by their staining characteristics, cytoplasmic granule size, T cell dependency, and expression of MC proteases and inflammatory mediators [7]. Briefly, mouse CTMCs express MC protease (Mcpt) 4, chymase 1 (Cma1), tryptase beta 2 (Tpsb2), tryptase alpha/beta 1 (Tpsab1), and carboxypeptidase A3 (Cpa3), but lack the MMC markers Mcpt1 and Mcpt2.

In several species, including human, the adult ovaries have different immune cells, such as macrophages, neutrophils, eosinophils, and MCs [8–11]. The appearance of these immune cells in the ovary is altered by the estrus cycle during the periovulatory period. It has also been suggested that MCs accumulate in mammary glands and uteri to mediate the structural reconstructions associated with altered sex hormones during the estrus cycle or pregnancy [12–14]. These reports indicate that MCs play some roles in reproductive functions. Furthermore, the appearance of MCs before sexual maturation has been shown in the ovaries of neonatal mice [15]. Although it has also been reported that a few MCs are present in the neonatal ovaries of C57BL/6 (B6) mice, the functional relationship between MCs and the perinatal ovary is unclear [16].

MRL/MpJ (MRL) mice originated from B6 (0.3%), C3H/He (12.1%), AKR (12.6%), and LG/J (75.0%) mice. MRL mice and their mutant strain, MRL/MpJ-*lpr/lpr* mice, are models for autoimmune diseases that resemble human systemic lupus erythematosus and rheumatoid arthritis [17,18]. In addition to autoimmune phenotypes, MRL mice show some unique phenotypes related to wound healing, such as accelerated ear punch closure and cardiomyocyte regeneration [19–21]. In addition, we have reported that the reproductive organs of MRL mice have other unique characteristics: specifically, metaphase-specific apoptosis of meiotic spermatocytes [22–24], heat shock resistance of spermatocytes found in experimental cryptorchidism [25,26], existence of testicular oocytes in newborn males [27–29], and development of ovarian cysts originating from the rete ovarii [30,31]. These phenotypes are closely associated with the genetic background of MRL mice, and several susceptibility loci have been identified by genomic analysis [21,24,26,29,31].

In the present study, we found that MRL mice possessed a greater abundance of MCs in the neonatal ovaries than other mouse strains. Furthermore, we determined that the ovarian MCs were mainly CTMCs and were in direct contact with the developing oocytes. We also found a correlation between the number of mast cells and the number of developing oocytes. Our findings suggest that the appearance of MCs in the neonatal ovary is influenced by the genomic background of the mouse strain and that ovarian MCs play a role in the regulation of early follicular development in MRL mice. 

## Materials and Methods

### Ethical statement

This study was approved by the Institutional Animal Care and Use Committee convened at the Graduate School of Veterinary Medicine, Hokkaido University (approval number: 11-0033). The investigators adhered to the Guide for the Care and Use of Laboratory Animals of Hokkaido University, Graduate School of Veterinary Medicine (approved by the Association for the Assessment and Accreditation of Laboratory Animal Care International).

### Animals

Outbred (ICR) and inbred (AKR, B6, BALB/c, CBA, C3H/He, DBA/1, DBA/2, NZB, NZW, and MRL) mouse strains were used in the present study. Eight- to ten-week-old male and female mice purchased from Japan SLC (Shizuoka, Japan) were maintained with free access to food and water at our facility. Timed mating was established by housing females with males overnight. At noon of the following day, females were checked for the presence of vaginal plugs, and the embryos were recorded as embryonic day 0.5 (E 0.5). The female MRL and B6 mice were examined from E15.5 to postnatal day 14 (P14). In addition, the male MRL and B6 mice and the females of the other strains were obtained at P0 to assess strain differences.

### Light microscopy

The liver, kidney, heart, spleen, skin, testis, and ovary from each mouse were fixed with 4% paraformaldehyde overnight, embedded in paraffin, cut into 1.5- to 3-µm-thick sections, and stained with hematoxylin and eosin (H&E) or toluidine blue (TB).

For histoplanimetry, the sections were stained with 1% TB in 70% ethanol for 30 min, and the number of MCs per organ was measured as the MC density (cells/mm^2^). In the ovaries, the MC density in the surface epithelial region, defined as the number of MCs in the area facing the surface epithelium per organ, was also measured.

### Immunohistochemistry

Immunohistochemistry was performed using the Histofine Mousestain kit (Nichirei, Tokyo, Japan) or the Histofine SAB-PO(M) kit (Nichirei) to detect Tpsab1, F4/80, CD3, and B220 as the cell markers for MCs, macrophages, T cells, and B cells, respectively. Each section was deparafﬁnized using xylene, rehydrated using graded ethanol, and washed with distilled water. Antigen retrieval was performed with 10 mM citrate buffer (pH 6.0) for 20 min at 105°C (Tpsab1 and CD3) or 0.1% pepsin for 5 min at 37°C (F4/80 and B220). The sections were treated with 0.3% hydrogen peroxidase/methanol solution for 30 min and incubated with blocking reagents (Nichirei) for Tpsab1 or normal goat serum for the other proteins. The sections were incubated at 4°C overnight with mouse anti-Tpsab1 antibody (1:200; Abcam, Cambridge, UK), rat anti-F4/80 antibody (1:100; Cedarlane, Ontario, Canada), rabbit anti-CD3 antibody (1:200; Nichirei), or rat anti-B220 antibody (1:1600, Cedarlane). The Tpsab1 sections were then treated with Histofine Simple Stain MAX-PO(M) (Nichirei) for 30 min. The other sections were treated with goat biotinylated anti-rabbit IgG antibody (Nichirei) for CD3 or goat biotinylated anti-rat IgG antibody (1:100; Life Technologies, Carlsbad, USA) for F4/80 and B220 for 30 min and then incubated with streptavidin-peroxidase (Nichirei) for 30 min. The immunopositive reactions were developed using a 3,3′-diaminobenzidine-H_2_O_2_ solution. The sections were also counterstained with hematoxylin.

### RT-PCR and quantitative real-time PCR

Total RNA from the ovaries of MRL and B6 mice at P0 was purified using TRIzol reagent (Life Technologies) and treated with DNase (Nippon Gene, Tokyo, Japan). cDNA was synthesized from the RNA using ReverTra Ace (Toyobo, Osaka, Japan) and random primers (Promega, Madison, USA). Each cDNA, adjusted to 1.0 µg/µL, was used for PCR with Go Taq (Promega) and the gene-specific primer pairs shown in Table 1. The amplified samples were electrophoresed using 1% agarose gel containing RedSafe (iNtRON Biotechnology, Kyungki-do, Korea) and photographed using a UV lamp. Quantitative real-time PCR (qPCR) analysis was performed using Brilliant SYBR Green QPCR Master Mix III and a real-time thermal cycler (MX 3000; Stratagene, Milano, Italy).

**Table 1:** Summary of the specific gene primers.

Gene	Forward Primer (5′–3′)	Reverse Primer (5′–3′)	Product size	Accession #
*Mcpt1*	AAACAGTCATAAATGGCAAG	GGGAACAAACCATCATCAC	237 bp	NM_008570
*Mcpt2*	GTGATGACTGCTGCACACTG	CTTGAAGAGTCTGACTCAGG	595 bp	NM_008571
*Mcpt4*	CCTTACATGGCCCATCT	CTTCCCCGGCTTGATA	327 bp	NM_010779
*Cpa1*	ACCTCTGCTGTGTGCTGGGATAG	TTTGCAGTTGACAATCTGGGTCTT	333 bp	NM_010780
*Tpsb2*	CTCTCTCATCCACCCACAGT	TGTAGATGCCAGGCTTGTTG	590 bp	NM_010781
	CGACATTGATAATGACGAGCCTC	ACAGGCTGTTTTCCACAATGG	80 bp	
*Tpsab1*	AGTGGCCAAGCCATTAGAG	TCTGGCTCACAGTCATCAGG	449 (351) bp	NM_031187
*Cpa3*	ACACAGGATCGAATGTGGAG	TAATGCAGGACTTCATGAGC	690 bp	NM_007753
*Tex101*	ATCTTTCTTCTAATCGCCTCACG	GCTCAGCCTTTGAAGTCCAGT	121 bp	NM_019981
*Bmp15*	CGATTGGAGCGAAAATGGTG	CCAGAGCTTCTGCTGAATAC	320 bp	NM_009757
*Gdf9*	ACGTATGCTACCAAAGAGGG	CAGAGTGTATAGCAAGACCG	204 bp	NM_008110
*Zp1*	CCCTGAGATTGGGTCAGCG	AGAGCAGTTATTCACCTCAAACC	164 bp	NM_009580
*Zp2*	GCACTTATGCTCTGGACTTGG	TGCTTGAATAGCTGGACAGAA	167 bp	NM_011775
*Zp3*	CCTCAGGACTAACCGTGTGGA	CCATCAGGCGAAGAGAGAAAG	149 bp	NM_011776
*Actb*	TGTTACCAACTGGGACGACA	GGGGTGTTGAAGGTCTCAAA	165 bp	NM_007393

For *Tpsab1*, C57BL/6 mice had a smaller gene product size than MRL/MpJ mice because of a 98-nucleotide deletion due to a point mutation at the exon 2/intron 2 splice site [32].

### In situ hybridization

The cRNA probes for *Mcpt2* and *Tpsb2* were synthesized in the presence of digoxigenin-labeled UTP using a DIG RNA labeling kit (Roche Diagnostics, Mannheim, Germany) to detect MMCs and CTMCs, respectively. Table 1 shows the primer pairs for each probe synthesis. Deparaffinized sections of the ovaries were treated with proteinase K and then incubated with hybridization buffer containing 40% formamide, 10 mM Tris-HCl (pH 7.6), 200 µg/mL RNA, 100 µg/mL DNA, Denhardt’s solution (Sigma-Aldrich, St. Louis, USA), 10% dextran sulfate, 600 mM NaCl, 0.25% SDS, and 1 mM EDTA (pH 8.0) for pre-hybridization. For hybridization, each section was incubated overnight with the sense or antisense RNA probe (final concentration: 0.3 µg/mL) in hybridization buffer at 58°C. After washing with SSC, the sections were incubated with sheep anti-digoxigenin Fab fragments conjugated to alkaline phosphatase (1:2000; Nucleic Acid Detection kit; Roche Diagnostics) for 6 h at room temperature. The signal was detected by incubating the sections with a color substrate solution (Roche Diagnostics) in a dark room overnight at room temperature. The sections were counterstained with nuclear fast red.

### Immunofluorescence

The deparaffinized sections were treated with 10 mM citrate buffer (pH 6.0) for 20 min at 105°C, treated with normal donkey serum, and incubated with mouse anti-Tpsab1 antibody (1:200) and rabbit anti-DEAD (Asp-Glu-Ala-Asp) box polypeptide 4 (DDX4) antibody (1:200; Abcam) for 3 h at room temperature or with mouse anti-Tpsab1 antibody (1:200) and rabbit anti-tumor necrosis factor-α (TNF-α) antibody (1:500; AbD Serotec, Oxfordshire, UK) at 4°C overnight. The sections were then incubated with Alexa Fluor 488-labeled donkey anti-mouse IgG and Alexa Fluor 594-labeled donkey anti-rabbit IgG secondary antibody (1:500; Life Technologies) for 30 min, followed by Hoechst33342 (1:200; Dojindo, Kumamoto, Japan) for 30 min. The immunoﬂuorescence signals were examined by confocal microscopy.

For histoplanimetry, the number of Tpsab1-positive MCs contacting DDX4-positive oocytes per ovary was calculated as the density of MCs contacting oocytes. Furthermore, the number of Tpsab1-positive MCs making contact with DDX4-positive oocytes per total number of Tpsab1-positive MCs was calculated as the ratio of MCs contacting oocytes. In addition, to evaluate early follicular development in mice, the numbers of DDX4-positive oocytes and the number of oocytes contacting Tpsab1-positive MCs were counted at each oocyte developmental stage. Briefly, the developmental stages of oocytes were categorized into 3 phases according to the morphology of the oocytes and follicles as follows: nest, several oocytes form cluster; primordial follicle, oocytes are surrounded by simple squamous epithelium; primary follicle, oocytes are surrounded by simple cuboidal epithelium. Intermediate follicles with a single granulosa layer that consisted of both flattened and cuboidal cells were scored as primary follicles in the present study.

### Electron microscopy

The ovaries of MRL and B6 mice at P0 were immediately fixed with 3% glutaraldehyde in 0.1 M cacodylate buffer (pH 7.3) at 4°C for 4 h. The ovaries were then post-fixed with 1% osmium tetroxide in 0.1 M cacodylate buffer at room temperature for 2 h, dehydrated with graded alcohol, and embedded in Quetol 812 (Nissin EM, Tokyo, Japan). Ultrathin sections were then stained with uranyl acetate and lead citrate and were observed using a transmission electron microscope.

### Statistical analysis

The results were expressed as mean ± SEM values and were analyzed using nonparametric methods. The Mann-Whitney *U* test was used to compare 2 groups. The Kruskal-Wallis test was used to compare 3 or more groups, and multiple comparisons were performed using Scheffé’s method. The correlation between 2 groups was analyzed by Pearson’s correlation test. 

## Results

### The appearance of ovarian MCs in neonatal MRL mice

**Figure 1:**
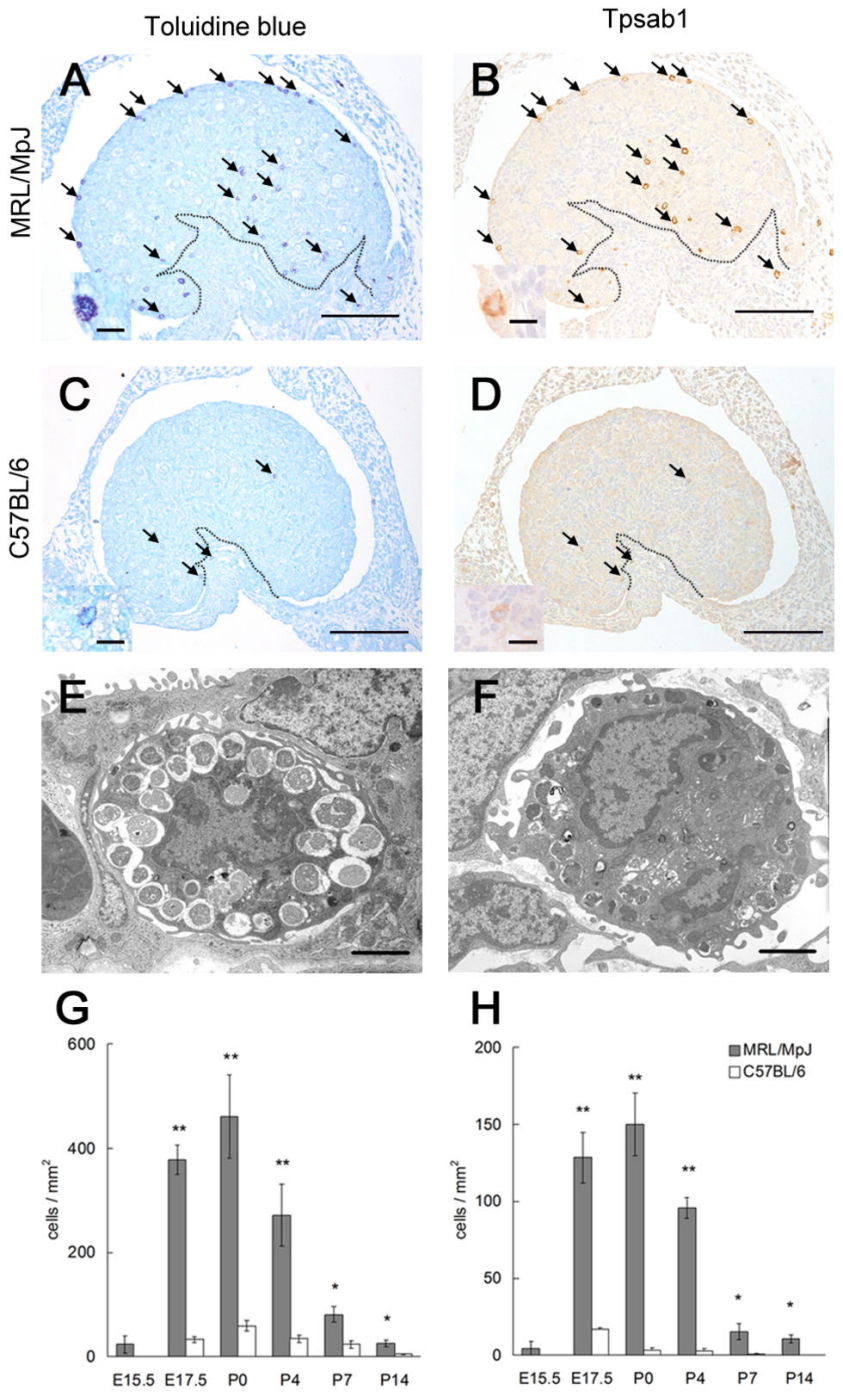
The appearance of mast cells in the ovaries of neonatal mice. (A–D) The ovaries of neonatal mice. Serial sections of mouse ovaries at postnatal day 0 were stained using toluidine blue (panels A and C) or immunohistochemistry for the mast cell (MC) marker Tpsab1 (panels B and D). In MRL/MpJ mice, numerous metachromatic MCs are observed in the cortex of the ovary (panel A, arrows), and these cells are positive for Tpsab1 (panel B, arrows). In C57BL/6 mice, a few metachromatic MCs and Tpsab1-positive cells are identified (panels C and D, arrows). The cytoplasmic granules showing metachromasy and a positive signal for Tpsab1 are more abundant and larger in MRL/MpJ mice than in C57BL/6 mice (panels A–D, insets). Arrows indicate the same cells. Dotted lines indicate the border between the cortex and medulla of the ovary. Bars in panels: 100 µm. Bars in insets: 10 µm. (E and F) The ultrastructure of MCs in mouse ovaries at postnatal day 0. The MCs of MRL/MpJ mice (panel E) have more and larger cytoplasmic granules than the MCs of C57BL/6 mice (panel F). The ovarian MCs have fold-like structures on the cell surface and segmented or non-segmented nuclei in both strains. Bars: 2 µm. (G and H) MC density in mouse ovaries. The number of metachromatic MCs in the whole area of the ovary (panel G) or in the surface epithelial region of the ovary (panel H) was divided by the area of the ovary. Data represent the mean ± SEM (n = 3–6 in each group). Significant differences between MRL/MpJ and C57BL/6 mice were analyzed using the Mann-Whitney *U* test at each time point. *: *P* < 0.05, **: *P* < 0.01.

At P0, TB staining revealed that the MRL mouse ovaries contained numerous metachromatic cells (Figure 1A); these cells also had Tpsab1-positive granules in their cytoplasm (Figure 1B). These results clearly demonstrated that the ovaries of MRL mice contained MCs. The MCs in MRL mice mainly localized to the ovarian cortex rather than to the medulla, and especially accumulated around the surface epithelial region (Figure 1A and B). In contrast, B6 mice had few MCs in either the ovarian cortex or the medulla (Figure 1C and D). Ultrastructural analysis revealed that the ovarian MCs had fold-like structures on the cell surface and segmented or non-segmented nuclei in both strains (Figure 1E and F). In the cytoplasm, the ovarian MCs contained numerous large electron dense granules in MRL mice (Figure 1E), but those in B6 mice had relatively few and small granules (Figure 1F) in accordance with the observations made by TB staining and Tpsab1 immunohistochemistry (see insets of Figure 1A–D). To investigate the appearance of the MCs, the MC density in the TB-stained ovary sections was calculated from E15.5 to P14. In MRL mice, the ovarian MCs were observed from E15.5; and the MC density in the whole ovary area significantly increased at E17.5, peaked at P0, and gradually decreased from P0 to P14 (Figure 1G). Similar results were observed in the MC density in the surface epithelial region of the ovary. In particular, MRL mice showed 40-fold higher values than B6 mice at P0 (Figure 1H).

**Figure 2:**
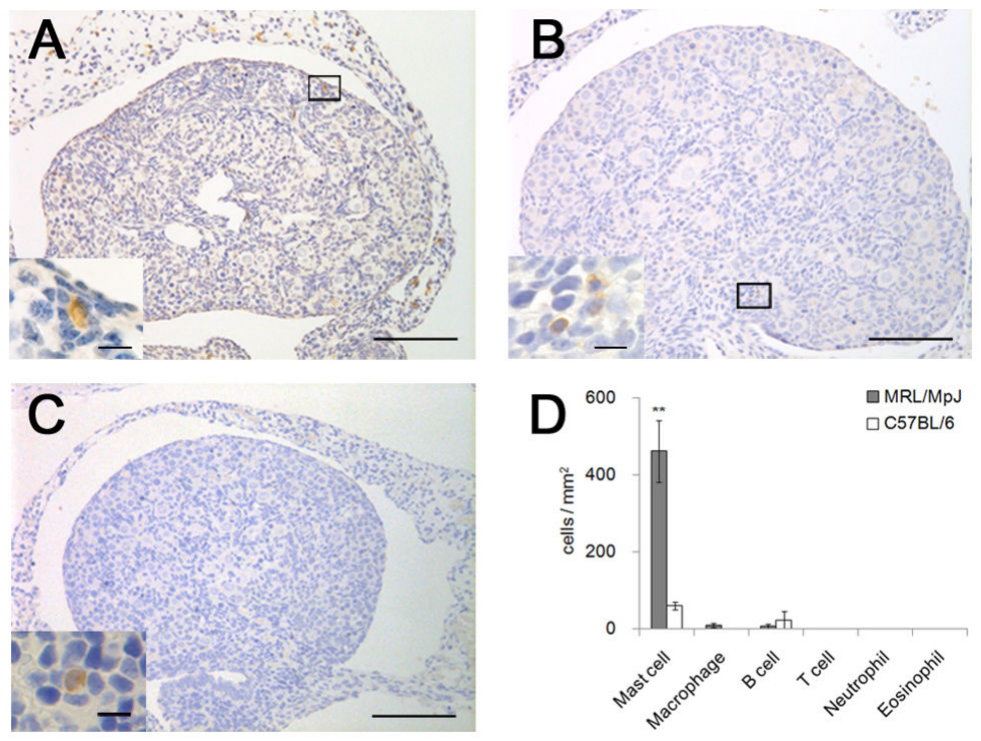
The appearance of immune cells in the ovaries of neonatal mice. (A–C) The ovaries of MRL/MpJ mice at postnatal day 0. Immunohistochemical analysis of F4/80 (macrophage marker, panel A), B220 (B cell marker, panel B), and CD3 (T cell marker, panel C). A few F4/80- and B220-positive cells (panels A and B), but no CD3-positive cells (panel C), are observed in the ovaries of the MRL/MpJ mice. The insets show a higher magnification of the boxed area in panels A and B. In panel C, the inset shows the spleen section at postnatal day 0 as a positive control. Bars in panels: 100 µm. Bars in insets: 10 µm. (D) Number of immune cells in the ovaries at postnatal day 0. Data represent the mean ± SEM (n = 3–6 in each group). Significant differences were analyzed using the Mann-Whitney *U* test. **: *P* < 0.01.

Immune cells such as macrophages and lymphocytes were rarely observed in the ovary sections of MRL and B6 mice at P0 (Figure 2A–D). In addition, H&E staining showed that neutrophils and eosinophils were not present in the ovaries of either strain (Figure 2D).

To examine whether numerous MCs were present in other organs, the MC densities in the liver, kidney, heart, spleen, skin, and testis were also analyzed in MRL and B6 mice at P0 (Table 2). MRL mice had higher numbers of MCs than B6 mice in all organs examined, but significant differences were only found in the female skin and ovaries and in the male liver and kidney. Interestingly, although MRL mice had greater MC density values in the ovary, only a few MCs were present in the testis. Thus, we confirmed that the appearance of numerous MCs was an ovary-specific characteristic in neonatal MRL mice.

**Table 2:** Mast cell density in various organs at postnatal day 0.

		Liver	Kidney	Heart	Spleen	Skin	Ovary/Testis
Female	MRL/MpJ	0.7 ± 0.1	2.9 ± 0.9	8.7 ± 1.1	575.8 ± 82.1	555.4 ± 19.2**	461.7 ± 79.8**
	C57BL/6	0.3 ± 0.1	1.0 ± 0.3	8.2 ± 0.6	372.6 ± 67.3	361.1 ± 25.0	59.2 ± 10.4
Male	MRL/MpJ	1.1 ± 0.3*	2.8 ± 0.2*	6.6 ± 1.1	658.4 ± 72.0	604.9 ± 66.9	4.1 ± 2.1
	C57BL/6	0.2 ± 0.2	0.2 ± 0.1	7.0 ± 1.0	356.1 ± 6.0	456.8 ± 32.2	0.0 ± 0.0

Units: number of mast cells/mm^2^. Data, shown as the mean ± SEM (n = 4–6 in each group), were analyzed using the Mann-Whitney *U* test. *P < 0.05, **P < 0.01.

### Strain-specific differences in the appearance of ovarian MCs in neonatal mice

**Figure 3:**
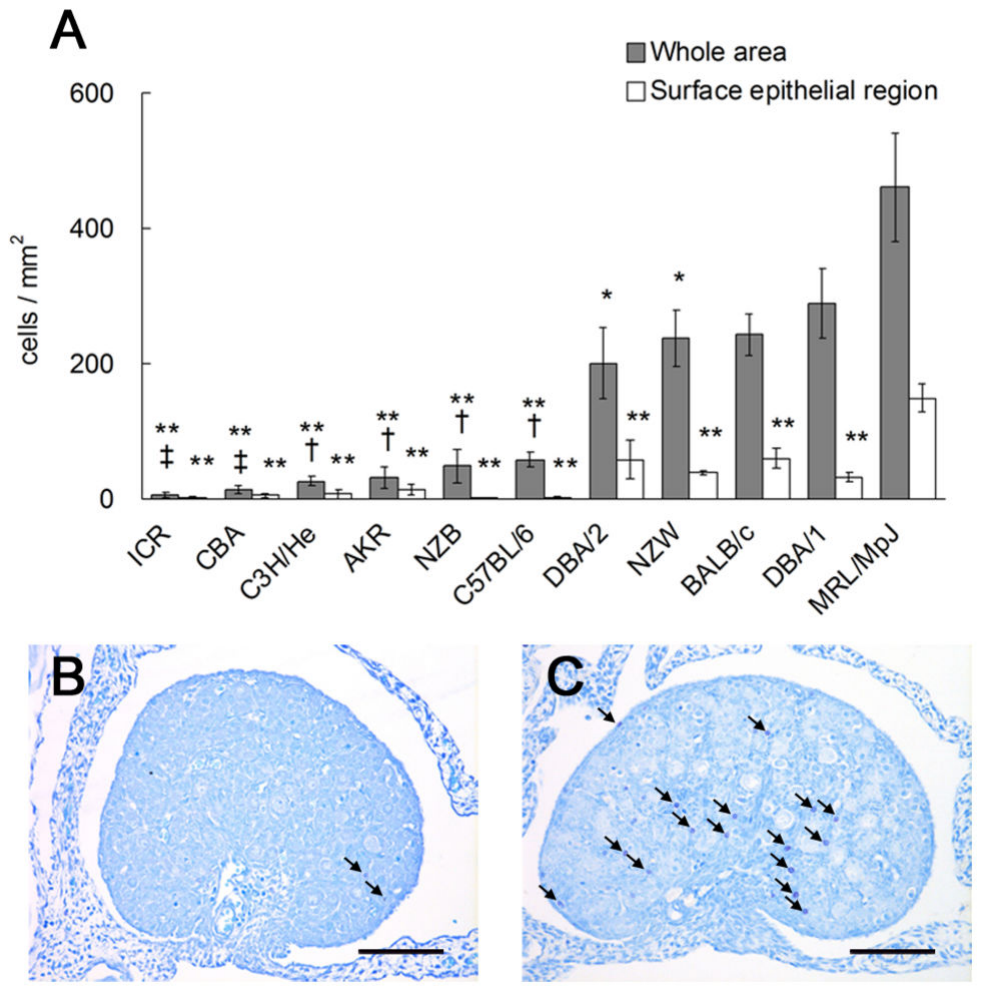
Strain differences in the appearance of ovarian mast cells in neonatal mice. (A) Ovarian mast cell (MC) density among 11 mouse strains at postnatal day 0. The number of metachromatic MCs in the whole area of the ovary (gray bars) or in the surface epithelial region of the ovary (white bars) was divided by the area of the ovary. Data represent the mean ± SEM (n = 4–8 in each group). Significant differences were analyzed using Scheffé’s method subsequent to the Kruskal-Wallis test. *: *P* < 0.05, **: *P* < 0.01 vs. MRL mice. †: *P* < 0.05, ‡: *P* < 0.01 vs. DBA/1 mice. (B and C) Toluidine blue-stained ovary sections of neonatal mice. Only a few ovarian MCs are present in CBA mice (panel B, arrows). BALB/c mice have metachromatic MCs mainly at the border between the cortex and medulla of the ovary (panel C, arrows). Bars: 100 µm.

To investigate whether the appearance of numerous ovarian MCs was a strain-speciﬁc phenotype of MRL mice, the ovarian MC density was compared among 11 mouse strains at P0. As shown in Figure 3, MRL mice had the greatest MC density among all the examined strains (Figure 3A). Comparatively, ICR, CBA, C3H/He, AKR, NZB, B6, and autoimmune-prone NZB mice possessed low MC densities (Figure 3A and B). Interestingly, the ancestral strains of the MRL mouse C3H/He, AKR, and B6 had significantly lower densities than MRL mice (Figure 3A). In addition, the MC densities of DBA/2, NZW, BALB/c, and DBA/1 mice were approximately half the value observed in MRL mice (Figure 3A); the MCs in these strains tended to appear in the cortex, but not in the surface epithelial region in the ovary, which was distinct from the localization in MRL mice (Figure 3C). Therefore, the appearance of numerous MCs and their localization to the surface epithelial region were strain-specific phenotypes in the ovaries of neonatal MRL mice.

### MC types in the ovaries of neonatal MRL mice

**Figure 4:**
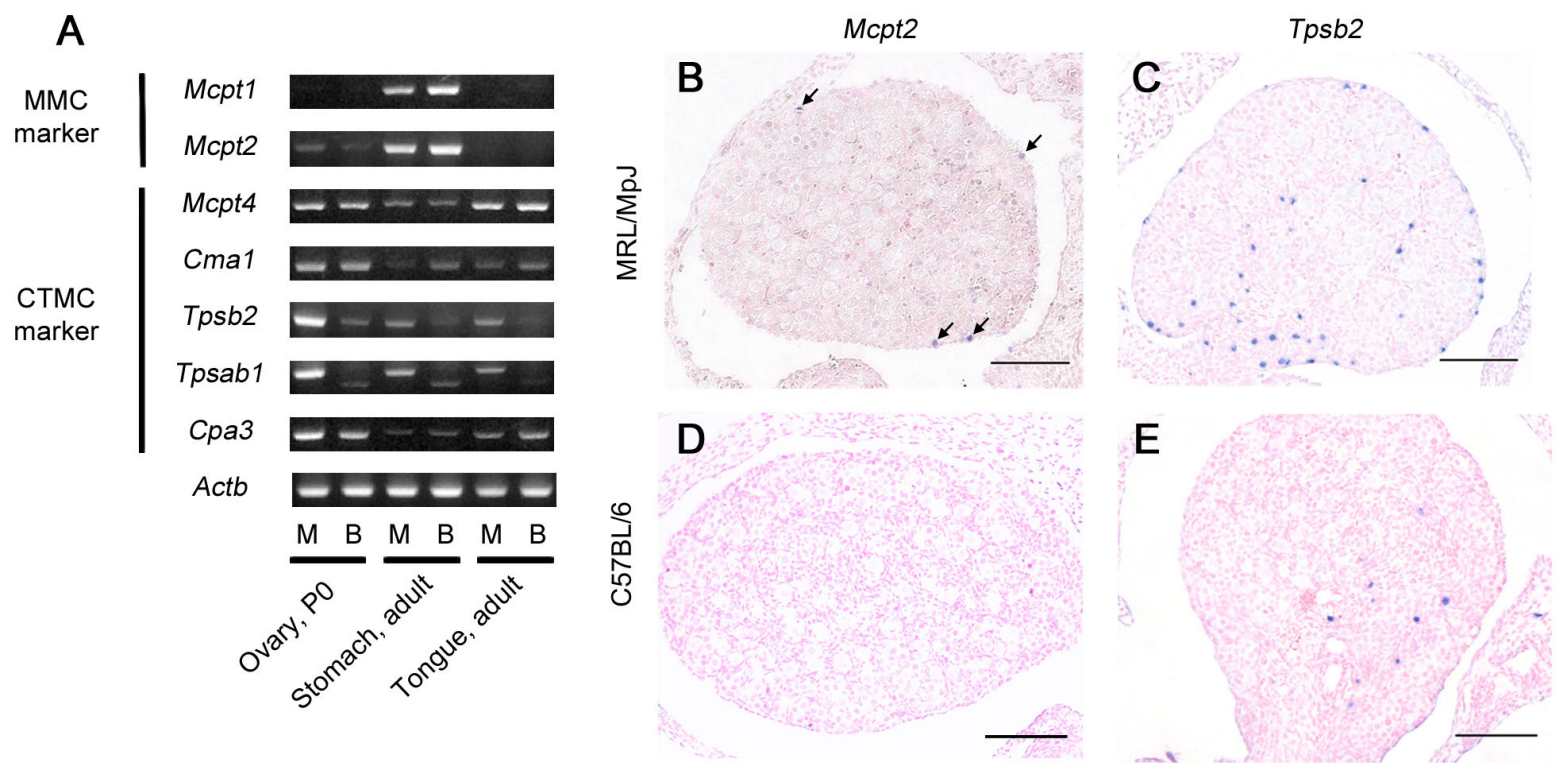
Gene expression of mast cell-specific proteases in the ovaries of neonatal mice. (A) RT-PCR was used to measure the mRNA expression of a mast cell (MC)-specific protease in the mouse ovary at postnatal day 0. Stomach and tongue samples from adult mice were used as positive controls for MMCs and CTMCs, respectively. *Actb* was used as an internal control. M: MRL/MpJ mice. B: C57BL/6 mice. (B–E) *In* *situ* hybridization of *Mcpt2* and *Tpsb2* in the ovaries at postnatal day 0. *Mcpt2*-positive signals are detected in the surface epithelial region of the MRL/MpJ mouse ovary (panel B, arrows). No positive signal is observed in the ovaries of C57BL/6 mice (panel D). *Tpsb2*-positive signals are detected in both strains (panels C and E) but are more abundant in MRL/MpJ mice (panel C). Bars: 100 µm.

To determine the MC type in the ovaries of neonatal MRL mice, the expression of MMC markers (*Mcpt1* and *Mcpt2*) and CTMC markers (*Mcpt4*, *Cma1*, *Tpsb2*, *Tpsab1*, and *Cpa3*) was examined by RT-PCR at P0 (Figure 4A). Weak *Mcpt2* expression was detected in the ovaries of MRL and B6 mice, and the band intensity of *Mcpt2* in MRL mice was slightly stronger than that in B6 mice (Figure 4A). No *Mcpt1* expression was detected in the ovaries of either strain. All the examined CTMC markers were detected in the ovaries of both strains. Although the band intensities of *Mcpt4* and *Cma1* in B6 and MRL mice were similar, the *Tpsb2*, *Tpsab1*, and *Cpa3* bands were stronger in MRL mice than in B6 mice (Figure 4A). The product size of *Tpsab1* differed between B6 and MRL mice because B6 mice have a 98-nucleotide deletion due to the point mutation at the exon 2/intron 2 splice site [32].

Furthermore, we used *in situ* hybridization to examine the mRNA expression of an MMC marker (*Mcpt2*) and CTMC marker (*Tpsb2*) in the ovaries of MRL and B6 mice at P0. *Mcpt2* mRNA was detected only in the ovaries of MRL mice (Figure 4B), and the *Mcpt2*-positive cells localized to the surface epithelial region of the ovary. However, no positive reaction was detected in B6 mice (Figure 4D). Comparatively, *Tpsb2* mRNA was detected in the ovaries of both strains, but the *Tpsb2*-positive cells were more abundant in MRL mice, especially in the surface epithelial region of the ovary, than in B6 mice (Figure 4C and E). Consequently, the numerous MCs in the ovary of neonatal MRL mice were considered CTMCs rather than MMCs.

### The relationship between ovarian MCs and oocytes

**Figure 5:**
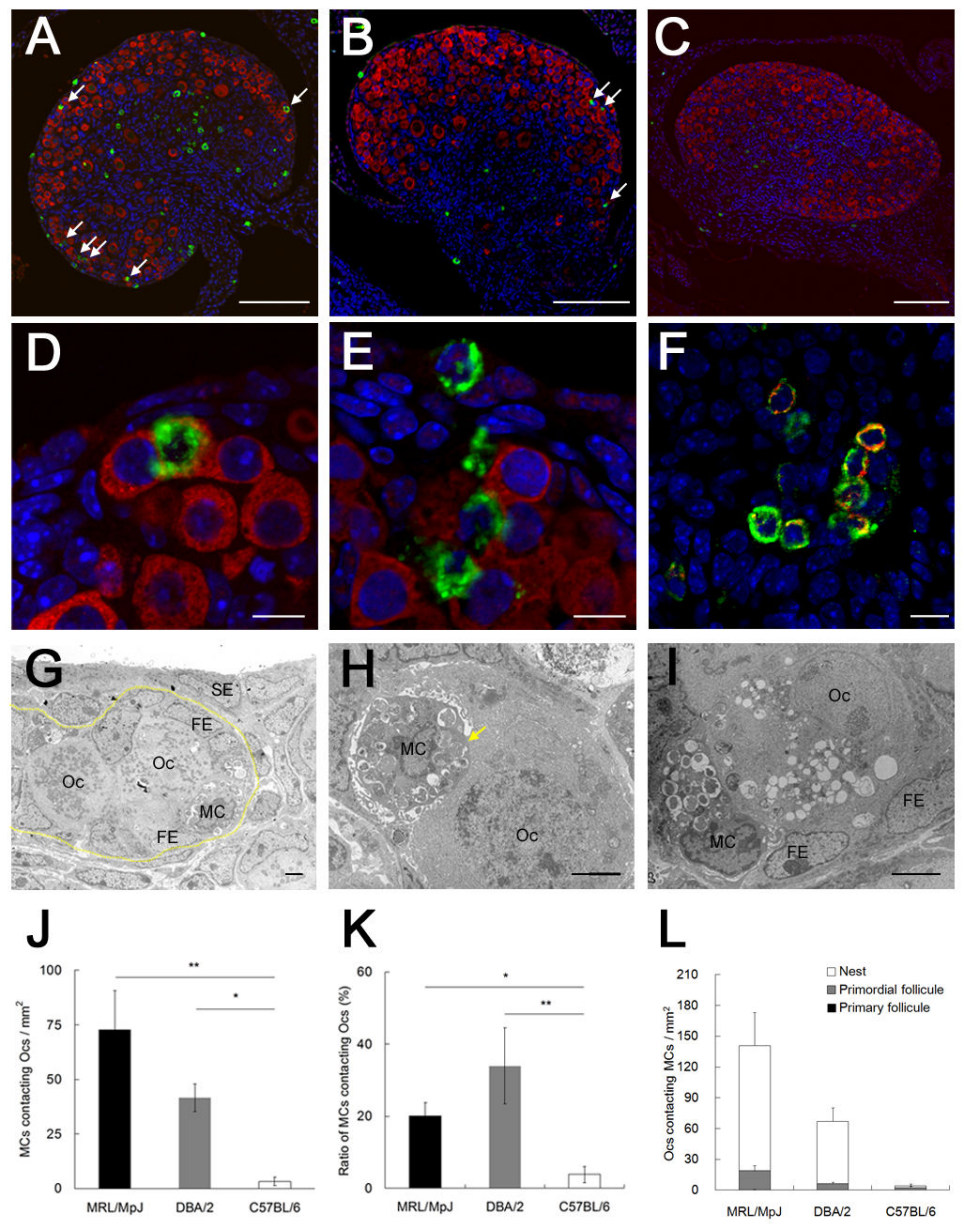
The relationship between mast cells and oocytes in the ovaries of neonatal mice. (A–C) The localization of mast cells (MCs) and oocytes (Ocs) in the ovary at postnatal day 0. Tpsab1 (green) and DDX4 (red) were detected as markers for MCs and Ocs, respectively. In MRL/MpJ (panel A) and DBA/2 (panel B) mice, Tpsab1-positive MCs are detected next to DDX4-positive Ocs (arrows), but only a few Tpsab1-positive signals are observed in C57BL/6 mice (panel C). Bars: 100 µm. (D and E) Higher magnification of the ovaries from MRL/MpJ and DBA/2 mice. In MRL/MpJ (panel D) and DBA/2 mice (panel E), some MCs contact the clusters of Ocs. In addition, some MCs compress the Ocs and show degranulated features. (F) The expression of TNF-α in the ovarian MCs of MRL/MpJ mice at postnatal day 0. TNF-α (red) is expressed in Tpsab1 (green)-positive MCs. Bars: 10 µm. (G–H) Ultrastructure of MCs and Ocs in the ovaries of neonatal MRL/MpJ mice. The nest (surrounded by a yellow dotted line), containing several Ocs and follicular epithelial cells (FEs), is observed beneath the surface epithelial cells (SEs), and MCs are observed in the nest (panel G). Parts of the Oc cytoplasm are compressed by MCs (panel H, arrow), and some Ocs in contact with MCs exhibit vacuolated cytoplasm (panel I). Bars: 2 µm. (J) The total number of MCs contacting Ocs. (K) The ratio of the number of MCs contacting Ocs to the total number of MCs. (L) The number of Ocs contacting MCs. Ocs were categorized according to the developmental stage: nest, primordial follicle, or primary follicle. Data represent the mean ± SEM (n = 4–5 in each group). Significant differences were analyzed using Scheffé’s method subsequent to the Kruskal-Wallis test. *: *P* < 0.05, **: *P* < 0.01.

To investigate the relationship between ovarian MCs and oocytes, the expression of the MC marker Tpsab1 and oocyte marker DDX4 was detected by immunofluorescence at P0 in MRL, DBA/2, and B6 mice, which had high, middle, and low ovarian MC densities, respectively (Figure 3A). The DDX4-positive oocytes localized to the ovarian cortex and accumulated beneath the surface epithelium in MRL, DBA/2, and B6 mice (Figure 5A–C). Interestingly, some Tpsab1-positive MCs were observed beside or within the cluster of DDX4-positive oocytes, and MCs showed degranulated features in MRL (Figure 5A and D) and DBA/2 mice (Figure 5B and E). MCs have various chemical mediators, such as TNF-α, whose levels increase with the maturation of MCs during fetal development [33]. In the MRL mouse ovary, the TNF-α signal overlapped that of Tpsab1, indicating the ovarian MCs produced TNF-α (Figure 5F).

In the neonatal mouse ovary, clusters of oocytes called nests break into smaller cysts and subsequently form primordial and primary follicles, respectively [34]. Ultrastructural analysis of MRL mouse ovaries revealed that some MCs directly contacted several oocytes forming large clusters in nests (Figure 5G). In addition, the cytoplasm of the oocytes was compressed by the MCs (Figure 5H), and some of the oocytes showed deformed or vacuolated structures (Figure 5I).

To quantify the relationship between MCs and oocytes, the density of MCs contacting oocytes (Figure 5J) and the ratio of MCs contacting oocytes (Figure 5K) were evaluated. The density of MCs contacting oocytes was highest in MRL mice, and the values obtained from MRL and DBA/2 mice were significantly higher than those from B6 mice (Figure 5J). The ratio of MCs contacting oocytes was also significantly higher in MRL and DBA/2 mice than in B6 mice (Figure 5K). Next, we categorized the oocytes in contact with the MCs into 3 follicle developmental stages, nest, primordial follicle, and primary follicles, and compared the numbers among the 3 strains. The number of oocytes contacting MCs was highest in MRL mice, whereas only a few oocytes contacted MCs in B6 mice (Figure 5L). In MRL and DBA/2 mice, most of the oocytes contacting the MCs formed the nest (Figure 5L).

**Figure 6:**
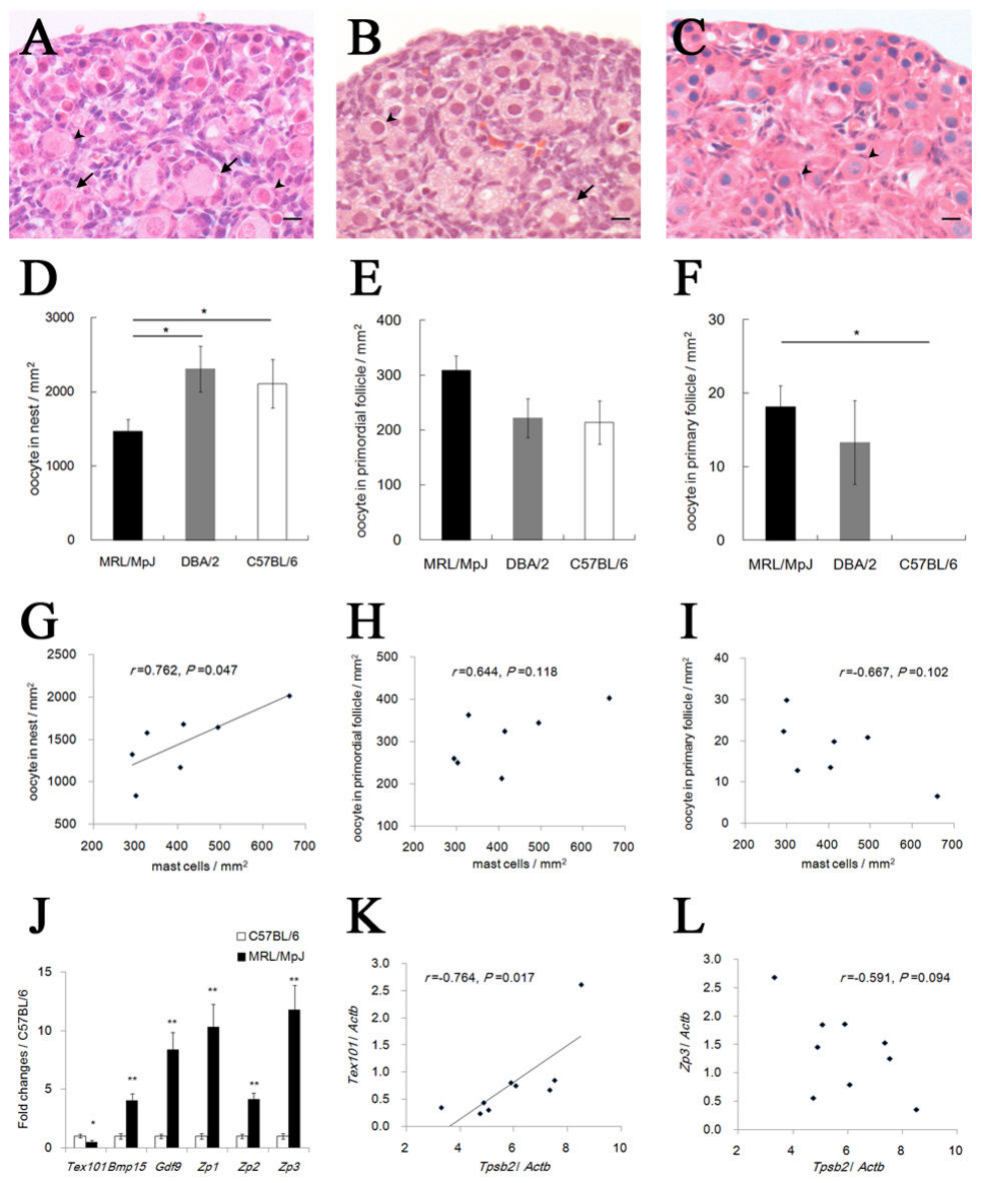
Early follicular development in the ovary of neonatal mice. (A–C) Mouse ovaries at postnatal day 0. Sections from MRL/MpJ (panel A), DBA/2 (panel B), and C57BL/6 (panel C) mice were stained using hematoxylin and eosin. In all strains, the oocytes just beneath the surface epithelium are found in large clusters called nests, whereas some oocytes are enclosed in primordial follicles in the deep cortex (panels A-C, arrowheads). In addition, MRL/MpJ (panel A) and DBA/2 (panel B) mice contain a few primary follicles in the deep cortex (arrows). Bars: 10 µm. (D–F) The number of oocytes in the mouse ovaries at postnatal day 0. DDX4-positive oocytes per ovary area were counted according to 3 developmental stages: nest (panel D), primordial follicle (panel E), and primary follicle (panel F). Data represent the mean ± SEM (n = 4–7 in each group). (G–I) Correlation between the number of mast cells and oocytes in the nest (panel G), primordial follicle (panel H), and primary follicle (panel I) in MRL mice at postnatal day 0 (Pearson’s correlation test, n = 7). (J) qPCR analyses of *Tex101*, *Bmp15*, *Gdf9*, *Zp1*, *Zp2*, and *Zp3* genes at postnatal day 0. Data represent the mean ± SEM (n = 7–9 in each group). (K and L) Correlation between the gene expression of *Tpsb2* and *Tex101* (panel K) or *Zp3* (panel L) in MRL mice at postnatal day 0 (Pearson’s correlation test, n = 9). Significant differences were analyzed using Scheffé’s method subsequent to the Kruskal-Wallis test (D–F) or Mann-Whitney *U* test (J). *: *P* < 0.05, **: *P* < 0.01.

To investigate the functional relationship between the appearance of ovarian MCs and early follicular development in neonatal mice, the follicle developmental stages were compared among MRL, DBA/2, and B6 mice. In all mice examined at P0, the oocytes just beneath the surface epithelium formed nests, whereas some oocytes in the deep cortex were enclosed by follicular epithelial cells (Figure 6A–C). In all mice, most of the follicular epithelial cells were squamous, but some were cuboidal in MRL and DBA/2 mice. To confirm these observations, the oocyte density was measured in every follicle developmental stage at P0 (Figure 6D–F). The density of nest-stage oocytes was the highest in all follicle stages (Figure 6D, compare the y-axes of Figure 6E and F), and MRL mice showed a significantly lower value when compared with DBA/2 and B6 mice (Figure 6D). There was no significant strain difference in the density of the primordial follicle (Figure 6E). On the other hand, the density of the primary follicle was significantly higher in MRL mice than in B6 mice (Figure 6F). In MRL mice at P0, the density of ovarian MCs significantly and positively correlated with the density of nest-stage oocytes, but not with the density of the primordial follicle (Figure 6G and H). The density of the primary follicle tended to negatively correlate with the density of MCs (Figure 6I). In Figure 6J, the expression of several genes relating to early follicular development was compared between MRL and B6 mice. In MRL mice, the expression of *Testis-expressed gene 101* (*Tex101*) was significantly lower, while the expression of *Bone morphogenetic protein 15* (*Bmp15*), Growth differentiation factor 9 (Gdf9), *Zona pellucida glycoprotein 1* (*Zp1*), *Zp2*, and *Zp3* was significantly higher (Figure 6J). Furthermore, the expression of *Tpsb2* derived from ovarian MCs showed a significant positive correlation with the expression of *Tex101*, expressed in nest-stage oocytes, and showed a trend of negative correlation with the expression of *Zp3*, expressed in the primary follicle [35,36] (Figure 4B, 6K, and 6L). 

## Discussion

### Abundant MCs in the ovary of neonatal MRL mice

MCs in neonatal ovaries were first reported in ICR mice [15]. The MCs were present in the hilus of the ovary, the mesovarium, and ovarian bursa, and were most abundant at P0, with numbers decreasing during the first postnatal week [15]. A few MCs have also been reported in the ovaries of neonatal B6 mice [16]. In the present study, we demonstrated that not only ICR and B6 mice, but also other strains possessed ovarian MCs at the neonatal stage. In the neonatal stage, MRL mice had the highest ovarian MC density, and the ovarian MCs principally localized to the surface epithelial region, which was distinct from the other strains. Interestingly, the analysis of MCs in various other organs suggested that the appearance of numerous MCs was an ovary-specific phenotype in neonatal MRL mice. Therefore, our results demonstrate for the first time that the appearance and localization of ovarian MCs is dependent on the mouse strain. Specifically, the abundance of ovarian MCs around the surface epithelial region is a novel and unique phenotype of neonatal MRL mice. We previously reported that the reproductive organs of MRL mice have unique characteristics that are controlled by several factors derived from the genomic background of this strain [21,24,26,29,31]. Although the genomic background could be one of the factors controlling the appearance of ovarian MCs, the ancestral strains of MRL mice, namely AKR, C3H/He, and B6 mice, had lower MC densities than MRL mice. From these findings, we propose that the appearance of MCs in the neonatal ovary is caused by strain-specific factors from LG mice or more than 2 of the ancestral strains or by epigenetic or environmental factors.

### The characteristics of ovarian MCs in neonatal MRL mice

Although basophils have metachromatic granules and mono- and multi-globular nuclei similar to MCs, basophils do not express MC-specific proteases [37,38]. In the present study, the metachromatic cells in the TB-stained ovary of neonatal MRL mice were immunopositive for Tpsab1, an MC-specific protease. Furthermore, immune cells other than MCs, such as T cells, B cells, macrophages, neutrophils, and eosinophils, were rarely observed in the ovaries of neonatal MRL mice. These results indicate that MCs are the only immune cells abundantly present in the ovary of neonatal MRL mice. Furthermore, electron microscopy results demonstrated that the ovarian MCs in neonatal MRL mice contained more granules than those in B6 mice. Because the size and number of cytoplasmic granules increase as MCs mature during fetal development [33,37], the ultrastructural characteristics of the MCs in MRL and B6 mice suggest mature and immature features, respectively.

All examined CTMC markers were detected in the ovaries of neonatal MRL and B6 mice. Interestingly, the expression of *Tpsb2* and *Tpsab1* was stronger in MRL mice than in B6 mice. Furthermore, in the surface epithelial region of the ovaries, *Tpsb2* (CTMC marker gene)-expressing cells were more abundant in MRL mice than in B6 mice. These results demonstrate that the majority of ovarian MCs identified are CTMCs. In addition, a small number of *Mcpt2* (MMC marker gene)-expressing cells were detected in the surface epithelial region of the ovaries from neonatal MRL mice, but not in those from neonatal B6 mice. The expression pattern of MC proteases differs across mouse strains and tissues. Briefly, although *Mcpt2* (MMC marker gene) was detected in the ear of WB/ReJ mice, no expression was detected in BALB/c mice [39]. Furthermore, CTMCs expressed both CTMC and MMC markers in the trachea and large bronchi of normal mice [40]. Therefore, these data emphasize that MMC marker-expressing MCs and abundant ovarian CTMCs are unique phenotypes in neonatal MRL mice. We propose that the mouse strain or ovary-specific microenvironment mediates the expression of MC-specific protease genes.

### The relationship between the appearance of ovarian MCs and early follicular development

In neonatal mouse ovaries, oocytes undergo a process called nest breakdown in which oocytes in the nest break into smaller cysts until a few individual oocytes remain. The individual oocytes are finally surrounded by follicular epithelial cells to form primordial follicles and then develop into primary follicles [34]. To determine the relationship between MCs and oocytes in the neonatal ovary, we observed the co-localization of MCs and oocytes with follicular development. In MRL and DBA/2 mice, more than 20% of the MCs localized mainly beside oocytes in the nest. Furthermore, the density of nest-stage oocytes and the gene expression of *Tex101* in nest-stage oocytes were significantly lower in MRL mice than in B6 mice. In addition, the density of primary follicles and the expression of genes associated with follicular development (*Bmp15*, *Gdf9*, *Zp1*, *Zp2*, and *Zp3*) were higher in MRL mice than in B6 mice. These findings indicate that MCs tend to localize beside the nest-stage oocyte rather than the primordial or primary follicle and that follicular development is faster in MRL than in B6 mice, which have high and low ovarian MC densities, respectively.

In MRL mice at P0, the density of MCs positively correlated with the density of nest-stage oocytes. Similarly, the gene expression of an MC marker had a significant positive correlation with the expression of a marker for nest-stage oocytes. Further, a trend of negative correlation was suggested between the density of MCs and the density of the primary follicle and between an MC marker gene and a primary follicle marker gene although ovarian MCs did not contact the primary follicle. Importantly, the number of ovarian MCs in MRL mice significantly increased at E17.5, before nest breakdown; they were most evident at P0, the time of nest breakdown, and decreased thereafter. Therefore, our results indicate that ovarian MCs increase in accordance with the timing of nest breakdown and decrease with follicular development in neonatal MRL mice. Approximately two-thirds of oocytes die during nest breakdown by several possible mechanisms, including apoptosis and autophagic cell death [41–44]. TNF-α, which induces apoptosis and autophagy, plays an important role in the process of germ cell death in fetal/neonatal mice and rats [45–47]. In addition, MCs can induce endothelial cell apoptosis via TNF-α [48,49]. In fact, ovarian MCs in MRL mice expressed TNF-α and directly contacted the degenerative oocytes in the nest at P0. Some of the degenerative oocytes in MRL mice exhibited vacuolated cytoplasm, which is typical of autophagy [50]. Therefore, ovarian MCs are closely related to nest breakdown and this process might be regulated by oocytic death via several mediators, such as TNF-αderived from the numerous ovarian MCs. In addition, the increase of the primary follicle in MRL mice might be a result of the accelerated nest breakdown.

In conclusion, we have demonstrated that the appearance of numerous ovarian MCs is strain-dependent. Their abundance, localization, and gene expression is unique in neonatal MRL mice. We propose that MCs are involved in the regulation of early follicular development, especially nest breakdown, which predicts a new function of MCs. 
